# FAP senescence: a critical event in muscle regeneration

**DOI:** 10.1038/s41392-023-01411-w

**Published:** 2023-05-10

**Authors:** Xia Kang, Yuqi Gao, So-ichiro Fukada, Hongming Miao

**Affiliations:** 1 Department of Pathophysiology, College of High Altitude Military Medicine, Army Medical University, Chongqing, 400038 China; 2 Pancreatic Injury and Repair Key Laboratory of Sichuan Province, The General Hospital of Western Theater Command, Chengdu, Sichuan China; 3 Institute of Medicine and Equipment for High Altitude Region, College of High Altitude Military Medicine, Third Military Medical University (Army Medical University); Key Laboratory of Extreme Environmental Medicine, Ministry of Education of China; Key Laboratory of High Altitude Medicine, People’s Liberation Army, Chongqing, 400038 China; 4 Project for Muscle Stem Cell Biology, Graduate School of Pharmaceutical Sciences, Osaka University, Suita, Osaka Japan; 5 Jinfeng Laboratory, Chongqing, 401329 China

**Keywords:** Senescence, Muscle stem cells

**Fig. 1:**
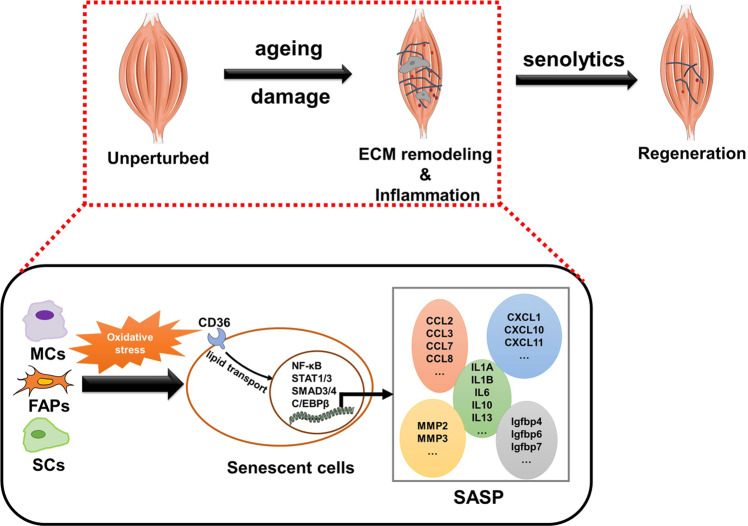
Schematic showing how senescent cells promote the formation of inflammatory and fibrotic stem cell niches in damaged muscles. During aging or damage, several cell types become senescent in response to oxidative stress, and the three major groups are MCs, FAPs and SCs. The upregulation of CD36 and enrichment of lipid transport pathways regulate the activation of inflammatory and fibrosis-associated transcription factors, thus stimulating the production of the SASP. The SASP facilitates inflammatory and fibrotic niche formation and inhibits the proliferation of muscle stem cells. MCs monocytes or macrophages; FAPs fibro/adipogenic progenitors; SCs satellite cells; SASP senescence-associated secretory phenotype; ECM extracellular matrix

In a recent study published in Nature, Moiseeva and colleagues examined senescent cells, including fibro/adipogenic progenitors (FAPs), muscle stem cells (MuSCs) and macrophages, which generate an inflammatory and fibrotic niche, thus inhibiting the activation of MuSCs and muscle regeneration.^1^ The researchers developed a method to distinguish endogenous senescent cells and showed the importance of cellular senescence as a potential target for improving muscle regeneration and repair (Fig. 1).^1^

FAPs are a group of mesenchymal stromal cells that specifically express Pdgfrα. In the last decade, numerous studies have revealed that FAPs are the dominant cell type that contribute to extracellular matrix (ECM) remodeling, leading to muscle fibrosis and fatty infiltration in muscle regeneration and degeneration. Moreover, FAPs showed a “double-edged sword” effect. These cells support the activation of MuSCs in the regenerative environment. In contrast, FAPs can also differentiate into adipocytes and myofibroblasts, thus leading to fibro-fatty degeneration in injured or atrophied muscles. In addition, FAPs could also impair the activation of MuSCs through the secretome in degenerative muscles.^2^ In general, the status of FAPs markedly regulates the stem cell niche.

Cellular senescence is permanent cell cycle arrest in dysfunctional cells, resulting in resistance to cell death.^1^ Senescence can be observed in both normal and abnormal conditions, including development, aging, and injury. In some conditions, senescence is beneficial to health. For example, the induction of senescence in tumor cells can prevent tumorigenesis.^3^ However, senescent cells may be harmful during tissue aging via their secretome, the so-called senescence-associated secretory phenotype (SASP). The SASP contributes to an inflammatory microenvironment, which can significantly impair tissue regeneration.^3^

Although several studies have revealed that FAP senescence can significantly impact muscle homeostasis, the role of senescent FAPs in muscle regeneration and repair remains controversial. Saito et al. showed that FAP senescence induced by exercise supported the regeneration of acutely injured muscles. The researchers found that exercise activated the AMPK pathway and induced FAP senescence. Moreover, senescent FAPs could be more easily cleared by macrophages, which is a critical stage in muscle regeneration.^4^ However, another study demonstrated that senescent FAPs in progeria-aged muscle negatively impacted the function of MuSCs. Muscle degeneration can be prevented by clearing senescent FAPs.^5^ These studies showed that enhancing the clearance of senescent FAPs was a promising way to promote muscle regeneration.

This study published in Nature verified that FAP senescence was not a positive event in muscle regeneration, even in young individuals.^1^ The authors established a technique to trace and isolate live senescent cells in vivo by labeling the classic senescence biomarkers p16^INK4a^ (encoded by the CDKN2A gene) and senescence-associated β-galactosidase (SA-β-gal) using p16-3MR mice and a fluorescent probe named SPIDER, respectively. The authors found that rare senescent cells were observed in uninjured muscles but were increased dramatically in the early stage of acute injury (3 days post injury) and decreased after 7 days post injury. This process was postponed in old mice. Next, the researchers found that cellular senescence was more severe in chronically injured muscle than in transiently injured muscles. These results indicated that senescent cells could be generated by injury and were positively associated with aging in individuals and the severity of injury.^1^ Consistent with the findings in previous studies, the clearance of senescent cells can efficiently promote muscle regeneration, regardless of age. However, the authors also showed that the properties of senescent cells in young mice possessed the original features of nonsenescent cells. For example, the differentially expressed genes and enriched pathways in senescent FAPs were highly similar to those of nonsenescent FAPs; however, the enriched pathways in senescent cells resulted in new cellular behaviors in old mice, including the activation of macrophage-associated pathways and complement/coagulation cascades in senescent FAPs.^1^

Unfortunately, the underlying reasons for the paradoxical role of FAP senescence in muscle regeneration and repair are unclear. One possible explanation is that the properties of FAP senescence induced by exercise may be different from that of FAP senescence in aging, since the authors found that oxidative stress-induced lipotoxicity drove cellular senescence regulated by the upregulation of the fatty acid receptor CD36 (Fig. 1), and exercise could prevent oxidation and lipotoxicity.^1,6^ Another reason may be that senescence and cell death occur simultaneously in injured muscles, and the capacity of FAPs to promote muscle regeneration may be dependent on FAP death but not senescence.

Collectively, senescent cells, especially FAPs, can generate an inflammatory and fibrotic microenvironment, which is harmful to the activity of stem cells. Clearing senescent cells is a good strategy for muscle regeneration. Furthermore, additional attention should be given to FAPs, and the switch from senescence to apoptosis in FAPs could be a promising way to treat muscle degeneration.
